# Writhing Movements and Hypoglycemia in Moderate–Late Preterm Infants: A Prospective Cohort Study

**DOI:** 10.3390/children12020174

**Published:** 2025-01-30

**Authors:** Javier Merino-Andrés, Francisco Javier Fernández-Rego, Álvaro Hidalgo-Robles, María Cayeiro-Marín, Purificación López-Muñoz, Soraya Pérez-Nombela

**Affiliations:** 1 Toledo Physical Therapy Research Group (GIFTO), Faculty of Physical Therapy and Nursing, University of Castilla-La Mancha, 45071 Toledo, Spain; javier.merino@uclm.es (J.M.-A.); soraya.perez@uclm.es (S.P.-N.); 2 Toledo Physiotherapy Research Group (GIFTO), Instituto de Investigación Sanitaria de Castilla-La Mancha (IDISCAM), 45071 Toledo, Spain; 3 Physiotherapy Department, University of Murcia, El Palmar, 30120 Murcia, Spain; 4 Early Intervention Research Group (GIAT), University of Murcia, 30100 Murcia, Spain; 5 Faculty of Education, International University of La Rioja, 26006 Logroño, Spain; alvaro.hidalgo@unir.net; 6 Centro Crecer, 45007 Toledo, Spain; maria.cayeiro@centrocrecer.es; 7 ImproveLab, Research in Pediatric Physiotherapy and Neurology Group, Faculty of Physical Therapy and Nursing, University of Castilla-La Mancha, 45071 Toledo, Spain; purificacion.lopez@uclm.es

**Keywords:** hypoglycemia, moderate–late preterm, general movement assessment, writhing movements

## Abstract

**Introduction:** This study aims to examine the relationship between hypoglycemia and general movement patterns during the early post-term-aged in moderate-to-late preterm infants and to assess the interobserver reliability of movement evaluation during this period. Compared with full-term infants, moderate-to-late preterm infants constitute the largest group of premature births globally and are at greater risk of developing neurodevelopmental disorders. Hypoglycemia is one of the most prevalent risk factors in this group. **Methods:** This prospective single-center explorative cohort study included moderate-to-late preterm infants during their admission in the neonatal intensive care unit. General movements were assessed via Prechtl’s General Movements Assessment, and blood glucose levels were monitored via the FreeStyle Optium™ Neo glucometer. Associations were analyzed via Fisher’s exact test, whereas interobserver reliability was evaluated via the intraclass correlation coefficient (ICC) and the kappa coefficient. **Results:** A total of 17 moderate-to-late preterm infants with hypoglycemia (<45 mg/dL) presented a relatively high percentage (58.8%) of poor repertoire and normal (35.2%) general movement patterns during the early post-term-aged. Interobserver reliability was good (ICC = 0.7), and the kappa coefficient indicated moderate reliability (0.4). **Conclusions:** Moderate-to-late preterm infants with transient hypoglycemia may frequently display poor repertoire movement patterns, highlighting the need for careful monitoring. Furthermore, the evaluation of general movements proves to be a reliable tool during the early post-term-aged.

## 1. Introduction

Moderate-to-late preterm infants (MLPTs), born between 32 weeks and 6 days and 36 weeks and 6 days of gestation, constitute the largest subgroup of preterm newborns, making up nearly 84% of all preterm births (PTs) [1]. Of these, approximately 72% are classified as late preterm infants, born between 34 weeks and 6 days and 36 weeks and 6 days of gestation [1,2,3]. This subgroup represents the majority of preterm births, with their incidence increasing significantly in recent years [3].

Preterm (PT) infants exhibit developmental differences compared to full-term newborns [1,4]. They show impairments in the motor [4,5], language [4,5], and cognitive domains [4,5,6,7]; have double the risk of neurodevelopmental disorders by the age of two [8]; and are more susceptible to neurological conditions, such as cerebral palsy (CP) [9], attention deficit hyperactivity disorder [10], and autism spectrum disorder (ASD) [10]. These outcomes are primarily attributed to a higher prevalence of neurological risk factors in this population [1]. Consequently, it is essential to prioritise evaluation, follow-up, and, when needed, interventions to support neurodevelopment, particularly for PT infants at higher gestational ages, counteracting the tendency toward non-monitoring [2].

Among these risk factors, the primary one affecting the most individuals is low gestational age [11]. However, this factor, along with others, increases the risk of neurological disorders. These risk factors can be neurological, such as intraventricular hemorrhage or periventricular leukomalacia, or non-neurological, such as hypoglycemia, hyperbilirubinemia, sepsis, or bronchopulmonary dysplasia [1,12,13,14].

The moderate–late preterm (MLPT) group has a lower prevalence of large-scale brain lesions and therefore fewer neurological risk factors compared with PTs born at less than 32 weeks of gestation [15,16], resulting in better outcomes compared with PTs born at less than 32 weeks of gestation [17]. Consequently, it makes sense to study non-neurological risk factors, as certain factors, such as hypoglycemia, hyperbilirubinemia, or sepsis, can lead to the development of CP in 10–18% of cases within this population group [18]. Thus, all risk factors can contribute to neurological impairment.

Hypoglycemia is one of the most prevalent factors, affecting 16% of moderate PTs and 8% of late PTs. The risk is greater when hypoglycemia is associated with other conditions, such as respiratory distress syndrome, hypothermia, eating disorders, hyperbilirubinemia, or intrauterine growth restriction [5]. The latter combination is particularly concerning, as it increases the likelihood of admission and prolonged admissions in the neonatal intensive care unit (NICU) [17].

This situation of hypoglycemia can negatively influence neurodevelopment in both PT and full-term newborns [3]. The neurodevelopmental repercussions are still uncertain, as there is only consensus in cases of severe hypoglycemia [19]. Some studies have shown increased incidence of developmental delay [20,21,22], fine motor impairment [20,21,22,23], and neurosensory disturbances [20,21,22], while other publications indicate that these developmental disorders may not be evident even at two years of age [20].

The causes of hypoglycemia in newborns are primarily associated with inadequate enteral intake, poor coordination of sucking and swallowing, delays or inefficiencies in oral feeding, and underlying conditions such as stress or sepsis. These factors are compounded by compensatory mechanisms at the metabolic level [17] due to the delayed activity of glucose-6-phosphatase [24]. If hypoglycemia is not effectively resolved, it may result in brain damage, although the specific threshold for this remains undefined [17]. The minimum blood glucose level that can cause short- and long-term neurological damage is still uncertain, though recent evidence suggests it may be lower than previously believed [19,22]. To mitigate risks, periodic monitoring is recommended, particularly when clinical symptoms of hypoglycemia are present. The American Academy of Pediatrics defines hypoglycemia as a blood glucose concentration below 47 mg/dL, while a threshold of 45 mg/dL is globally recommended during the first 48 h of life [19,25].

It is recommended to screen for high-risk neurological infants (a term used when clinical suspicion exists but a definitive diagnosis cannot yet be made) [11]. Infants with altered glucose levels should be assessed using evidence-based tools, such as the General Movement Assessment (GMA), the Hammersmith Infant Neurological Examination (HINE), and magnetic resonance imaging for early detection. These measures facilitate timely interventions for infants at high neurological risk [11,26,27]. Screening should be guided by clinical history and risk factors, including neonatal hypoglycemia, and initiated as early as possible, even in the NICU [11,26]. Evidence suggests that evaluating general movements during the writhing period (from preterm to 6 weeks post-term) in moderate-to-late preterm infants (MLPTs) can identify abnormal patterns in 75% of moderate preterms and 68% of late preterms [27]. The normal pattern is characterized by limb movements of small to moderate amplitude and slow to moderate speed, with an elliptical shape that creates the impression of writhing [28]. Additionally, suboptimal scores on the HINE and the NICU Network Neurobehavioral Scale during this period are associated with delayed neurodevelopment [27,29].

The use of early detection tools has made it possible to address certain neurodevelopmental disorders, such as hyperbilirubinemia and minor neurological impairments, without requiring the maximum severity of a risk factor or a high accumulation of specific substances [30]. As a result, countries like the Netherlands have started adapting their neonatal care strategies to prevent the development of these conditions. It is recommended to implement regular follow-ups through health and social policies, not only for extreme preterm infants but also for moderate-to-late preterm infants (MLPTs), as they constitute the largest group [31].

The primary aim of this study was to investigate the relationship between hypoglycemia and general movement patterns (GMs) during the early postnatal writhing period (corresponding to the early post-term age) in the MLPT group. A secondary aim was to evaluate the interobserver reliability of GMs during this period within the same population.

## 2. Methods

This was a prospective cohort study to investigate the relationship between hypoglycemia and GM patterns during the early post-term age and the interobserver reliability within this period of the GMA. This study was approved by the Clinical Research Ethics Committee of the Nuestra Señora del Prado Hospital in Talavera de Reina (33/2018) and by the Clinical Research Ethics Committee of the Virgen de la Salud Hospital in Toledo (426/2019). This study was registered at ClinicalTrials.gov with the following registration number: NCT04073836.

### 2.1. Participants

The subjects included in this study were MLPTs (subjects born at 32^0/6^ weeks to 36^6/6^ weeks of gestation) who were admitted to the NICU or intermediate care units at the Hospital Nuestra Señora del Prado de Talavera de la Reina or the Hospital Virgen de Salud de Toledo, both in Castilla la Macha (Spain), between 2018 and 2021 and whose parents agreed to provide informed consent. The exclusion criterion included MLPTs who were prescribed the following medications: barbiturates, antiepileptics, indomethacin, or dexamethasone.

The convenience sample was divided on the basis of the blood glucose levels measured within the first 48 h of life, with an exposed group (MLPTs with glucose levels below 45 mg/dL) and an unexposed group (MLPTs with glucose levels above 45 mg/dL). According to the global recommendation, we used the blood glucose parameter of 45 mg per deciliter as the cut-off [19,25]. The treatment protocol applied for subjects with hypoglycemia was as follows. In the case of detecting hypoglycemia between 25 and 45 mg/dL, nutritional support was given using a bottle based on weight: 10–15 mL for a weight less than 2000 g, 15–20 mL for a weight between 2000 and 25,000 g, 20–25 mL for a weight between 2500 and 3500 g and 25 mL for a weight greater than 4000 g. In the case of detecting hypoglycemia less than 25 mg/dL, IV glucose was given using the following formula: 6 g/kg/day.

### 2.2. Instruments and Evaluation Procedure

Hypoglycemia was assessed using capillary heel swabs from the newborn, tested with a FreeStyle Optium™ Neo glucometer (Abbott Diabetes Care Inc., Alameda, CA, USA). The first blood samples were taken 30 to 60 min after birth and then at regular intervals before feeding (depending on the values and clinical status). The blood glucose level measurements were carried out by the NICU nursing staff. The treatments applied for the subjects suffering from hypoglycemia were breastfeeding and IV glucose administration.

Prechtl’s GMA is an objective, valid, and reliable method [32]. GMs from preterm to 6 weeks post-term are called writhing movements [28]. They are characterised by the variability of movements, a small to moderate amplitude, and a slow to moderate speed [28]. The types of abnormal GMs in the writhing period are movements of poor repertoire (PR), cramped synchronised (CS), and chaotic (CH). The PR pattern is observed when there is a lack of variability, characterized by a monotonous movements that lack complexity. The CS pattern shows rigid movement that lacks smoothness and fluidity, which are characteristic features of the normal pattern. In this pattern, all the muscles of the limbs and trunk contract and relax almost simultaneously [32].

Prechtl’s GMA was performed by recording 10 min of video of each PT during their stay in the NICU or intermediate care unit within each gestational week once the medical stabilization of each subject was achieved and the neonatologist approved it. These recordings were acquired by means of a GoPro Hero 7™ camera (GoPro, San Mateo, CA, USA), which was placed at the foot of the crib using a tripod, recording each subject positioned in a supine position. Within the period studied, the recordings were taken either in the infants’ wakeful or sleep phase but always during the early morning hours, when the environment was free from external stimuli and without the performance of any intervention procedures. This recording methodology was applied to all infants included in this study. The videos were examined by two evaluators (M.C.-M. and J.M.-A.) blinded with respect to the hypoglycemia level of each subject. In the event of discrepancies between the evaluators, a third blinded evaluator (Á.H.-R.) made the final decision. All three evaluators had previously obtained basic certification in general movements. The last recording prior to the discharge of each subject was the recording analyzed at a statistical level.

### 2.3. Statistical Analysis

Descriptive analysis of the demographic variables was carried out using percentages and frequencies. To study their homogeneity, a Chi-square test was performed. In addition, analysis of the different categorical variables was carried out to investigate the possible associations between them, for which Fisher’s exact test was used. To analyze the quantitative variables, ANOVA of one factor was carried out once the assumptions of normality and homoscedasticity were verified. In all studies, the type one error level was set at *p* < 0.05.

Furthermore, backward stepwise logistic regression was performed to identify potential predictor variables for the different GMs patterns and different risk factors. This allowed us to determine which risk factors might exert a more significant impact on GM patterns within the MLPT population. In each step of this regression, the *p*-value was used as the criterion for variable selection, and the Akaike information criterion converged.

Interobserver agreement was calculated on the basis of the videos of GMs during the early postnatal writhing period. Interobserver agreement was determined on the basis of the evaluations of both examiners, and the intraclass correlation coefficients (ICCs) for the categories of general movements in the early post-term age were calculated. According to Shrout et al. [33], the following values are established: an ICC ≤ 0.4 indicates weak agreement; an ICC between 0.4 and 0.6 is moderate, an ICC between 0.6 and 0.8 is good, and an ICC > 0.8 is excellent. The confidence interval was 95%, with values of *p* < 0.05 considered significant. In addition, interobserver reliability was calculated using Cohen’s kappa coefficient [34]. According to Landis and Koch [35], the kappa coefficient can be classified as follows: below 0 indicates poor agreement, from 0 to 0.2 is light, from 0.2 to 0.4 is reasonable, from 0.4 to 0.6 is moderate, from 0.6 to 0.8 is substantial, and from 0.8 to 1 is almost perfect.

All analyses were performed with R version 4.0.3 (R Core Team 2020, R Foundation for Statistical Computing, Vienna, Austria).

## 3. Results

### 3.1. Demographics

**Figure 1:**
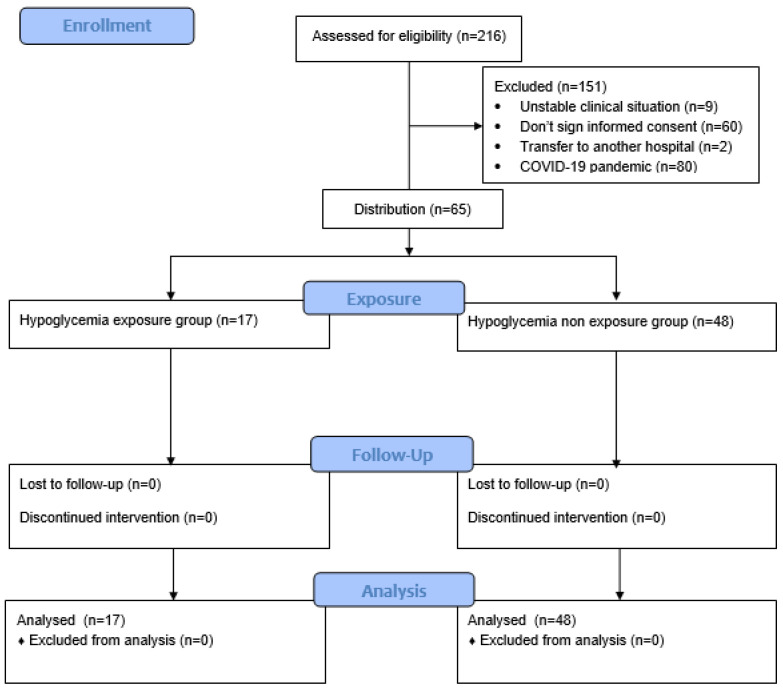
Flowchart diagram of the inclusion process.

Within the inclusion period, the families of 216 MLPTs were informed of this study, in which 65 babies (30.1%) were included. The reasons for excluding subjects included the impossibility of recording due to an unstable clinical picture (*n* = 9, 4.1%), the parents or main caregivers not signing the informed consent (*n* = 60, 27.7%), transfer to another hospital (*n* = 2, 0.9%), and hospital admission during the COVID-19 pandemic (*n* = 80, 37%). No subjects were excluded for barbiturates, antiepileptics, indomethacin, or dexamethasone. Figure 1 presents a flowchart diagram of the inclusion process.

The gestational age for the exposed group was 34.5 ± 1.1 weeks, the birth weight was 2140 ± 301.9 g, the length at birth was 43.5 ± 1.9 cm, and the head circumference was 31.3 ± 3.5 cm. The gestational age for the unexposed group was 34.3 ± 1.3 weeks, the birth weight was 2267.2 ± 421.7 g, the length at birth was 44.3 ± 3.9 cm, and the head circumference was 31.7 ± 1.4 cm, where the only significant difference between the groups was the amount of glucose in the blood (Table 1). The distribution by sex in the exposed group was 41.1% (*n* = 7) girls, whereas that in the unexposed group was 45.8% (*n* = 22). Within the exposed group, 23.5% (*n* = 4) of the subjects attended some early intervention services, whereas 16.6% (*n* = 8) of the unexposed group attended these services. The exposed group presented 47% (*n* = 8) of twin births, whereas the unexposed group presented 25% (*n* = 12). After the different Z scores (weight, length, and head circumference) were analyzed, no significant differences were found between the groups, despite lower values of the Z score for length in the exposed group. A total of four small gestational age infants were obtained, of which only one suffered from hypoglycemia, whereas a total of seven large gestational age infants were obtained, of which only one suffered from hypoglycemia. Finally, 20 subjects were born in multiple births, 8 of whom had hypoglycemia. The characteristics of the patients are shown in Table 1.

Regarding the distribution of the total sample on the basis of perinatal risk factors, we found a high percentage of risk factors typical of this population group, including hyperbilirubinemia (36.9%), hypoglycemia (33.8%), and respiratory alterations (30.7%) (Table 2). Among the total number of subjects included in this study, 17 did not present any risk factors, with 3 subjects in the exposed group and 14 in the unexposed group. After performing backward stepwise logistic regression, only one relationship was found between the use of ventilation and possible development of the CS pattern [36].

### 3.2. Hypoglycemia and General Movements

The exposed group consisted of 17 subjects whose blood glucose values were lower than 45 mg/dL. The unexposed group consisted of 48 subjects whose blood glucose values were above 45 mg/dL. The mean glucose level in the exposed group was 33 ± 7.3 mg/dL, whereas in the unexposed group, it was 69.05 ± 13.3 mg/dL. The treatment of choice to improve the hypoglycemia of the subjects in the exposed group was the administration of IV glucose (*n* = 12), while the remaining subjects improved this clinical sign through food (breastfeeding) (*n* = 5). No subject presented persistent hypoglycemia. The demographic data of the premature population with a diagnosis of hypoglycemia are presented in Table 3.

**Figure 2:**
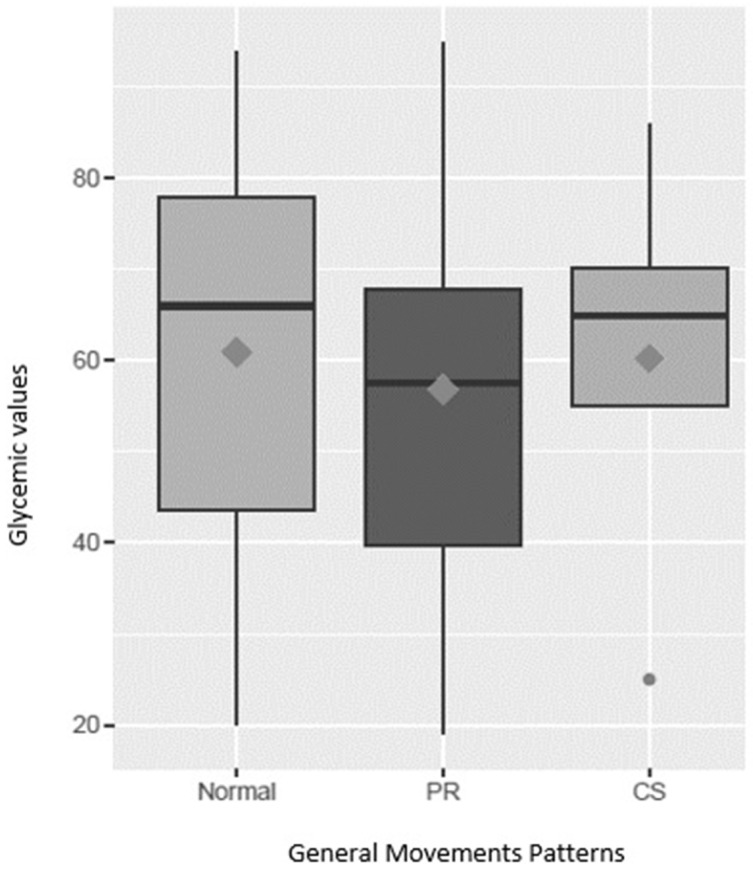
Glucose values according to the general movement patterns. The figure shows the maximum, minimum, and median values of the blood glucose values of the three groups of movement patterns during the early post-term age of the entire population analyzed.

Within the GM patterns during the early postnatal writhing period, the following distribution was found for the exposed group: 6 subjects exhibited a normal pattern (35.2%), 10 subjects exhibited a PR pattern (58.8%), and 1 subject exhibited a CS pattern (5.8%). The distribution of the control group was as follows: 21 subjects exhibited a normal pattern (43.7%), 24 subjects exhibited a PR pattern (50%), and 3 subjects exhibited a CS pattern (6.2%). Within the exposed group, 12 subjects were within the limits between 30 mg/dL and 45 mg/dL, where the distribution of the patterns was 5 subjects with a normal pattern and 7 subjects with a PR pattern. With respect to the five subjects who had less than 30 mg/dL, four had a PR pattern, and one had a CS pattern. The recordings for the evaluation of the GMs were taken at an average of 8.77 days of life for the exposed group (Table 4 and Figure 2).

Looking at the total number of subjects and their distribution by general movement patterns during their early postnatal writhing period, it can be seen that 40% normal patterns (*n* = 26), 53.8% PR patterns (*n* = 35), 6.1% CS patterns (*n* = 4), and no CH patterns were obtained. Of the total sample, 7.6% of those with normal patterns (*n* = 2), 34.6% of those with PR patterns (*n* = 9), and 25% (*n* = 1) of those with CS patterns (*n* = 1) received some early intervention.

### 3.3. Reliability

The results of the interobserver agreement in the early post-term-age indicate a good degree of agreement (ICC = 0.7; 95% CI = 0.6–0.8) between the two main evaluators (M.C.-M. and J.M.-A.). The result of the interclass subcategory agreement (N, PR, CS, and Ch) between the two main examiners was 70.6% (65 of 92). In terms of the average agreement on the trajectory of these subjects, based on more than one video during their admission in the NICU, a result of 52.3 (11 of 21) was found. The kappa coefficient indicates moderate reliability (0.4) between the primary examiners. Among all the babies, 67.6% had only one gross movement recording within the analyzed period (Table 5).

## 4. Discussion

Our study revealed that infants with transient hypoglycemia exhibited a high percentage of PR movement patterns (58.8%) and normal movement patterns (35.2%). Additionally, these subjects had low percentages (23.5%) of follow-up and surveillance of their neurodevelopment by the various services involved in the early detection and intervention of children at neurological risk. Regarding the interobserver reliability of the GMs, this study demonstrated a good degree of agreement (ICC = 0.7) and moderate reliability (0.4).

With hypoglycemia as a risk factor and its relationship with GMs, Nogolová et al. [5] studied the relationship between neonatal hypoglycemia and GMs in a population of PT and full-term newborns. These authors reported a total of 36 children (72% of the total sample) with abnormal GM patterns. During the early postnatal writhing period, the most frequently described movement pattern was PR, observed in 34 children (68% of all abnormal patterns), while a CS pattern was seen in only two children (4% of all abnormal patterns), and no chaotic movement patterns were found [5]. A normal writhing movement pattern was observed in 14 children (28% of the total sample). Finally, during the fidgety period, the absence of a fidgety movement pattern was observed in only nine children [5].

Our results align with those of Nogolová et al.; in both cases, a PR pattern was more frequently observed during the writhing period of the GMs. However, their study included all population groups of PTs, whereas our study did not follow up during the fidgety period, which has been shown to be more predictive than writhing period patterns. Subjects with blood glucose levels lower than 30 mg/dL exhibited worse movement patterns. Therefore, the continuous monitoring of PTs with hypoglycemia [23] who present abnormal GM patterns during their writhing period is recommended, as some of these individuals may develop neurodevelopmental disorders. Early detection and intervention are crucial, as the largest therapeutic window is during the first years of life [37]. During this period, various recovery processes are activated through neurogenesis after nervous system injury [38], which can be modulated through activity-dependent interventions capable of synaptic modification [39]. The minimum blood glucose level that can cause short- and long-term neurological damage remains unknown, although it appears to be lower than previously thought [19,22]. Therefore, periodic monitoring of these subjects is recommended.

Among the results obtained for the GMs in the MLPT population, our findings indicate a high percentage of PR patterns compared to the other patterns during the writhing period. This contrasts with the results obtained by Mehler et al. [40], who found almost all normal patterns (65/67) in the first assessment of the GMs during the writhing period, and 100% normal patterns (62/62) in the second assessment [40]. This disparity may be due to the fact that the vast majority of their sample presented a low risk (less than 5) on the Nursery Neurobiologic Risk Score instrument, indicating fewer risk factors in their study [40]. This aspect could be similar in our population, as they also presented few risk factors and we implemented early interventions to reduce exposure to them.

Within the population of late PTs, Brogna et al. [41] studied 574 subjects using various assessment instruments, including GMs. They reported 472 normal GM patterns at one month of corrected age (82%), 74 PR patterns (13%), 21 CS patterns (4%), and 7 CH patterns (1%) [41]. A total of 87% of the subjects obtained a normal diagnostic result, whereas 13% presented neurodevelopmental abnormalities at 24 months of corrected age, as assessed by the Bayley Scales of Infant Development. Of these, 9% had slightly abnormal development and 4% were diagnosed with CP. These results differ from those obtained in our study.

This disparity between studies may be related to several variables to consider in the population of newborn PTs. The first variable is the timing of the recording used for the evaluation of GMs, as it has been verified that the PR movement pattern is initially the most frequent and tends to normalize after a few weeks [42,43]. The second variable is the number of risk factors each subject experiences. Kerstjens et al. [22] studied neonatal comorbidities and their implications for neurodevelopment in moderate PTs, finding developmental delays in preschool-aged children when hypoglycemia was combined with asphyxia during the neonatal period [22]. The third variable is the number of recordings made of each subject, as there is a moderate relationship with neurodevelopmental outcomes at 14 months [44], which is insufficient for early diagnosis. Therefore, it is necessary to use more early detection instruments [45], which is another variable that may have influenced the results of our study compared to others. Another variable to consider is the duration of exposure to hypoglycemia and the comorbidity of risk factors each subject may experience [23]. Finally, therapeutic measures for treating hypoglycemia can help reduce possible neurological damage [19,22], minimizing severe motor pathologies but not necessarily other minor neurodevelopmental repercussions minimised.

Interobserver reliability was assessed in this study based on the concordance between the two main evaluators using the kappa coefficient. Among the studies that analyzed the percentage of concordance, we found mixed results. For example, Crowle et al. reported an agreement of 66–77% [46] within the writhing period, while several studies found values above 80%, ranging from 83% [47] to 89% [22,48,49,50]. On the other hand, studies analyzing the kappa coefficient showed varying results. Some studies reported a kappa coefficient above 0.80 [28,41,49,50,51], indicating almost perfect agreement, while others found moderate agreement, with a kappa of 0.6 [47], or substantial agreement, with values between 0.6 and 0.8 [52]. The study by Aizawa et al. showed the greatest interval variability, with a kappa ranging from 0.5 to 0.9 [53].

Bernhardt et al. [54] analyzed interobserver reliability in both periods of GMs separately. Within the writhing period, at two measurement times, they obtained reasonable reliability in the first analysis and moderate reliability in the second. These results are in line with those obtained in this investigation. However, the authors recommended analyzing a complete trajectory within the writhing period to obtain optimal results for the assessment of prognosis through general movements [54].

Crowle et al. [55] conducted a study on newborns with heart disease who had undergone cardiac surgery and obtained 54% interrater agreement (101 subjects) during the writhing period. The authors reported that during the writhing period, many doubts arose between normal patterns and PR patterns, so the support of notes and the option of reaching an agreement increased interobserver reliability [55]; these aspects were also present in this investigation.

Both Crowle et al. and Bernhardt et al. noted that the writhing period is complex to evaluate due to the greater number of categories to be scored compared to the fidgety period. Additionally, children evaluated with a gestational age close to term present greater complexity in terms of evaluation [55]. Both authors observed that the videos obtained exhibited complex variability, differing from those used as models during the evaluators’ training period [54,55]. In many studies with high interobserver reliability, the evaluators have been certified trainers from GMTrust [55].

However, Ricci et al. [56] obtained 97% within-observer reliability by subcategory and 100% agreement in the trajectory of the GMs. The main differences between the two studies are that, in our case, 67.6% of the cases were evaluated with a single video, and the trajectory evaluated was only that included in the writhing period. Therefore, it is extremely important to carry out a complete trajectory of both periods of GMs to make a more reliable prognosis, especially for those subjects that show anomalous GM patterns.

### Limitations

The primary limitation of this study was the small sample size of the group exposed to hypoglycemia. Another limitation was the significant number of losses from the study due to the COVID-19 pandemic, as the research team could not visit hospitals to recruit subjects. Additionally, the glucometer used in the evaluation was not validated for the study population. Related to the previous limitation, we found that the recording of the GMA evaluation did not coincide with the periods of hypoglycemia. Another limitation was that the subjects were not followed through the fidgety period, which is more predictive of neurodevelopment than the writhing period. It would be necessary to obtain a greater number of recordings of each subject to compare both groups. Additionally, analyzing the videos depending on the hypoglycemic state and the normalized state of glycaemia would provide more information. Finally, data on various pathologies or the mother’s medication were not collected, which could affect the child’s neurodevelopment.

## 5. Conclusions

Transient neonatal hypoglycemia in the MLPT population may lead to a high frequency of the development of normal and PR patterns of GMs during the early post-term age within the GMA. The latter pattern should be followed by early intervention services to monitor the child’s neurodevelopment.

The interobserver reliability of the early post-term age of the GMs presented a good degree of agreement and moderate reliability, reflecting that the evaluation using GMs was a reliable instrument within this period.

## Figures and Tables

**Table 1:** Sample demographic data.

Variable	Exposed Group (*n* = 17)	Unexposed Group (*n* = 48)	*p*
**Gestational age (w)**	34.5 ± 1.1	34.3 ± 1.3	0.10
**Weight (g)**	2140 ± 301.9	2267.2 ± 421.7	0.10
**Weight Z score**	−0.6 ± 0.8	−0.2 ± 0.8	0.10
**Length (cm)**	43.5 ± 1.9	44.3 ± 3.9	0.20
**Length Z score**	−0.9 ± 1.1	−0.3 ± 1.4	0.09
**Head circumference (cm)**	31.3 ± 3.5	31.7 ± 1.4	0.10
**Head circumference Z score**	−0.5 ± 1.9	−0.5 ± 1.9	0.23
**Gender (M/F)**	10/7	26/22	-
**EIS (Yes/No)**	4/13	8/40	-
**Glycaemia (mg/dL)**	33 ± 7.3	69.05 ± 13.3	*

Means ± standard deviations. * *p* < 0.001. cm (centimeters); EIS (early intervention services); F (female); g (grams); M (male); w (weeks).

**Table 2:** Distribution of perinatal risk factors by group.

Variable *n* (%)	Exposed Group (*n* = 17)	Unexposed Group (*n* = 48)	*p*	Total Sample
**Apgar (at 5 min)**	0	1 (2)	-	1 (1.5)
**Intracranial hemorrhage (positive neuroimaging)**	0	1 (2)	-	1 (1.5)
**Periventricular leukomalacia (positive neuroimaging)**	3 (17.6)	4 (8.3)	0.5	7 (10.7)
**Other nervous system alterations (positive neuroimaging)**	3 (17.6)	4 (8.3)	0.5	7 (10.7)
**Weight less than the 10th percentile for gestational age**	1 (5.8)	3 (6.2)	-	4 (6.1)
**Dysmorphic traits**	0	2 (4.1)	-	2 (3)
**Ventilation (non-invasive)**	7 (41.1)	13 (27)	0.4	20 (30.7)
**Sepsis (positive blood culture)**	3 (17.6)	7 (14.5)	1	10 (15.3)
**Hyperbilirubinemia**	3 (17.6)	21 (43.7)	0.1	24 (36.9)
**Other medical problems**	3 (17.6)	11 (22.9)	0.9	14 (21.5)
**Apnea + bradycardia**	3 (17.6)	9 (18.7)	1	12 (18.4)
**Persistent arteriovenous ductus**	0	1 (2)	-	1 (1.5)

**Table 3:** Demographic data of the preterm exposed group.

Subject	Gender	Gestational Age (w + d)	Weight (g)	Weight Z Score	Length (cm)	Length Z Score	Head Circumference (cm)	Head Circumference Z Score	Glycaemia (mg/dL)	Treatment	GMA Score
**1**	F	35 + 1	2310	−0.2	45	−0.	31	−0.5	25	Breastfeeding	CS
**2**	M	34 + 6	1904	−1.3	40	−2.7	30.5	−1.1	38	Breastfeeding	N
**3**	M	36 + 0	1800	−2.1	43	−2	31	−1.2	42	IV Glucose	PR
**4**	M	35 + 0	2100	−0.9	45	−0.5	30.5	−1.1	19	IV Glucose	PR
**5**	M	34 + 5	1933	−1.1	42	−1.8	28.5	−2.4	39	Breastfeeding	PR
**6**	F	36 + 0	2050	−1.5	42	−2.4	34	1.4	39	Breastfeeding	PR
**7**	M	33 + 5	2160	0	45	0.1	30	−0.9	38	Breastfeeding	PR
**8**	M	35 + 5	3060	1	48.5	0.9	35	1.9	25	IV Glucose	PR
**9**	M	36 + 0	2380	−0.7	46	−0.5	32	−0.4	37	IV Glucose	N
**10**	M	36 + 0	2480	−0.5	48	0.5	35	1.8	27	IV Glucose	PR
**11**	F	35 + 5	2105	−1.2	43	−1.8	32	0	20	IV Glucose	N
**12**	F	35 + 6	2090	−1.3	NR	NR	NR	NR	40	IV Glucose	PR
**13**	F	35 + 4	1840	−1.8	43	−1.7	20	−6.1	38	IV Glucose	PR
**14**	F	33 + 0	1925	0.2	43	−0.2	31	0.4	39	IV Glucose	N
**15**	F	33 + 0	1928	0.2	43	−0.2	31	0.4	31	IV Glucose	N
**16**	M	33 + 0	2155	0.4	43	−0.3	30	−0.6	31	IV Glucose	N
**17**	M	34 + 0	2160	−0.1	42	−1.4	32	0.3	33	IV Glucose	PR

**No subject presented persistent hypoglycemia**. cm (centimeters); CS (cramped synchronized); d (day), F (female); g (grams); M (male); mg/dL (milligram/deciliter); N (normal); NR (not reported); PR (poor repertoire); w (weeks).

**Table 4:** List of patterns of GMs by group.

Writhing Pattern	Exposed Group (*n* = 17)	Unexposed Group (*n* = 48)	*p*
Normal	6	21	0.86
Poor repertoire	10	24	0.84
Cramped synchronized	1	3	1
Chaotic	0	0	-

**Table 5:** Interobserver reliability.

ICC	0.7
CI 95%	0.6–0.8
% agreement	70.6%
Kappa coefficient	0.4

CI, confidence interval; ICC, intraclass correlation coefficient.

## Data Availability

All data generated or analyzed during this study are included in this published article.
